# Implementing and Improving Bedside Teaching and Experiential Learning for Medical Students During Psychiatry Placements

**DOI:** 10.1192/bjo.2025.10270

**Published:** 2025-06-20

**Authors:** Joseph Farmer, James Gardner, James Bloomfield

**Affiliations:** Coventry and Warwickshire Partnership NHS Trust, Coventry, United Kingdom

## Abstract

**Aims:** The General Medical Council’s (GMC) outcomes for graduate doctors includes the completion and interpretation of a psychiatric history, mental state examination (MSE), risk assessment and cognitive examination. To achieve these outcomes, medical students rotate through specialist psychiatry placements during their undergraduate training. Psychiatry rotations typically involve observing doctors in ward rounds and clinics and attending classroom-based teaching sessions. Feedback from local medical students highlighted that there was limited opportunity to complete a full psychiatric history, including a risk assessment, and mental state examination. This Quality Improvement Project (QIP) aimed to 1. Develop a reliable and accessible sign-up process and 2. Improve medical student’s bedside teaching experience during their psychiatry placement.

**Methods:** A driver diagram was used to identify primary and secondary drivers and interventions relating to the aims of the project. An iterative four-stage problem-solving model, Plan-Do-Study-Act (PDSA) approach was used. Students were invited to join bedside teaching sessions in pairs, with students taking turns to complete a history including a risk assessment, and an MSE, followed by feedback from a facilitator. In total 74 students attended bedside teaching sessions. Facilitators were recruited on a voluntary basis and included both foundation doctors, general practice specialty trainees and core psychiatry trainees. Student experiences of both the sign-up process and confidence in core psychiatric skills were captured in pre- and post-teaching surveys consisting of free text responses and Likert scales. In total, eight PDSA cycles were completed with the feedback from each cycle used to refine the sign-up process and improve the bedside teaching experience.

**Results:** In total 74 students completed the pre-teaching surveys and 48 completed the post-teaching survey. Prior to bedside teaching sessions only 18% of students felt confident visiting an inpatient ward to take a history from a patient, 17% felt confident taking a psychiatric history and 27% felt confident completing an MSE. After attending bedside teaching sessions, 73% of students indicated that they felt confident visiting an inpatient ward, 85% felt confident in taking a psychiatric history, and 85% felt confident completing an MSE.

**Conclusion:** Providing medical students with dedicated bedside teaching sessions led to significant increases in confidence in spending time on inpatient wards, and in the GMC core graduate outcomes of eliciting a psychiatric history, risk assessment and completing an MSE.

